# Seed Antioxidants Interplay with Drought Stress Tolerance Indices in Chilli (*Capsicum annuum* L) Seedlings

**DOI:** 10.1155/2018/1605096

**Published:** 2018-05-15

**Authors:** U. Lakshmi Sahitya, M. S. R. Krishna, R. Sri Deepthi, G. Shiva Prasad, D. Peda Kasim

**Affiliations:** ^1^Department of Biotechnology, K L (Deemed to be University), Green Fields, Vaddeswaram, Guntur, Andhra Pradesh 522 502, India; ^2^Professor Jaya Shankar Telangana State Agricultural University, Rajendranagar, Hyderabad, India

## Abstract

Altering climatic conditions and water stress drastically affects the chilli crop yield. In this scenario we adapted a strategic approach for screening of elite chilli genotypes, by exploring role of seed antioxidants in stress tolerance during vegetative phase. A total of 20 chilli genotypes' seed antioxidant potential and its effect on water stress tolerance were studied at three water regimes, namely, control (100% Field Capacity), moderate (80% Field Capacity), and severe (60% Field Capacity) stress conditions. Drought tolerance traits relative water content, chlorophyll content, and activities of superoxide dismutase and catalase enzymes were measured. A strong correlation was observed between seed antioxidants and water stress tolerant traits in seedlings. Genotypes KCa-5, KCa-6, and KCa-10 showed low quantity of H_2_O_2_ and Malondialdehyde in seeds and maintained high membrane integrity and chlorophyll content in seedlings. High content of proline in KCa-5, KCa-7, and KCa-10 seeds retained high relative water content at seedling stage under severe water stress. Present work reveals genotypic differences of hot pepper to different water regimes. Based on Principal Component Analysis (PCA) of seed antioxidant variables and drought tolerance indices twenty genotypes segregated into three clusters, namely, drought tolerant and susceptible and moderately tolerant.

## 1. Introduction

Climate change, a global phenomenon, has an adverse effect on crop production. Increased reiteration of extreme weather is implicated in the rapid climate change [1]. These abiotic stress factors restrain plants from reaching out to their maximum potential, thereby limiting crop productivity. As an extreme event, drought has become a significant problem affecting global plant production. Alongside, intensification of anthropogenic activities eventuated in radical depletion of water availability for agricultural practices. By the year 2100, the frequency and intensity of drought may increase from 1% to 30% with respect to global warming, which is increasing at an alarming rate [2]. Globally drought has predominantly reduced maize (11.6%), wheat (9.2%), and soybean (33.1–12.2%) productions [3]. There exists distinctive drought trend and frequencies in different regions of India [4]. By the years 2050–2099, drought events were expected to project in west central, central northeast, and peninsular regions of India [5]. In India, effects of drought are exacerbated because of deviated monsoon [6], groundwater depletion [7], and increasing population [8].

Crop experiences drought when water transpiration rate exceeds with absorption rate or when the supply of water to the roots is interrupted. Plants respond and adapt to water stress invariably by complex mechanisms inducing various morphological, biochemical, physiological, and molecular aspects resulting in either drought avoidance or drought escape or drought tolerance and the mechanism is highly varied among the plant species [9]. Water stress results in the excessive production of Reactive Oxygen Species (ROS), leading to oxidative stress [10]. ROS in plant system leads to membrane damage and many other changes, eventually leading to programmed cell death [11]. Both enzymatic and nonenzymatic antioxidant defense mechanisms of the plant system coordinate and alleviate oxidative damage in cell. Thus plants with abundant antioxidants were considered to possess superior tolerance towards oxidative damage.

Chilli (*Capsicum annuum* L) is an important horticultural crop and it has huge diversity and cultivated widely for its pungent fruits. It is considered as a significant commercial crop due to its enriched antioxidants, high pungency, rich flavour, and vitamins. As stated by Lee and Kader [12], intake of 100 g fresh weight of pepper is equipped with 100–200% of recommended daily administration (RDA) of Vitamin C. It also comprehends many plant derived compounds exhibiting anti-inflammatory, antiallergic, anticarcinogenic and antioxidative properties [13]. Cultivation of pepper is mainly confined to warm and semiarid regions where irrigation becomes limiting factor often resulting in decreasing the yield [14]. Several studies reported that chilli yield was drastically reduced due to water stress [15].

Though many of protective mechanisms are housekeeping and get activated at time of stress, for true tolerance seed specific desiccation protection mechanisms are required. Growth and establishment of seedling are very crucial at vegetation period as they determine the growth at later stages in plant life cycle. Also, according to Bláha and Pazderu [16], seed traits determine the plant growth during vegetation period. This emphasizes the significance of seed antioxidants and their implication to stress tolerance during seedling stage. Saleh and Plieth [17] reported a close relationship between tolerance against drought stress and antioxidant activity. Furthermore, works reported that ability of abiotic stress tolerance can be evaluated at seed level by analyzing antioxidative potential of seeds in chickpea [18]. Besides, Illing et al. [19] reported tolerance in the vegetative tissues of plants towards stress is acquisition of desiccation tolerance from seeds. Because of multiplicity of factors in field conditions, attempts to determine degree of tolerance with a single parameter possess limited scope. Also, because of high interaction of plant with environment, there subsist genotypic differences within same plant. We hypothesized that hot pepper seed biochemical traits would correlate with water stress tolerance at seedling phase. In the present investigation, we studied the relation between chilli seed extract antioxidant potential and water stress tolerance at seedling phase.

## 2. Materials and Methods

### 2.1. Seed Material

Twenty elite chilli genotypes, developed at K L University, Guntur, Andhra Pradesh, India (Table 1), were used in this study. From each genotype, 3 g of seeds was grinded and sieved through a 100 *μ*m mesh to obtain fine powder.

### 2.2. Seed Antioxidant Assays

#### 2.2.1. Estimation of DPPH Radical Scavenging Activity

For the determination of radical scavenging activity, seed powder (100 mg) was mixed with 2 mL of methanol and incubated overnight at room temperature. One mL of filtrate was added to 3 mL of 0.1 mM DPPH and incubated at dark conditions for 30 min. Absorbance was read at 515 nm using spectrophotometer (Genesys 10-S, Thermo Fischer Scientific, Madison, WI, USA) and percentage of DPPH scavenging activity was determined as described by Okoh et al. [20] using the following formula:

(1) DPPH  Activity  %=Absorbance  of  control−Absorbance  of  sampleAbsorbance  of  control∗100.

#### 2.2.2. Determination of Reducing Power

To determine reducing power, chilli seed powder (500 mg) was mixed with 5 mL of methanol. To 1 mL of extract 5 mL of phosphate buffer (2 M, pH 6.6) and 5 mL of 1% potassium ferricyanide were added. Mixture was incubated at 50°C for 20 min and 5 mL of 10% trichloroacetic acid was added. Reaction mixture was centrifuged at 1252 ×g for 10 min. To 5 mL of supernatant, 5 mL of ddH_2_O and 1 mL of 0.1% ferric chloride were added. Absorbance was read at 700 nm using spectrophotometer (Genesys 10-S, Thermo Fischer Scientific, Madison, WI, USA). Reducing power of seed extracts was determined according to Do et al. [21].

#### 2.2.3. Extraction and Estimation of Proline

Proline was estimated as described by Sankar et al. [22]. Chilli seed powder (500 mg) was extracted with 10 mL of 3% sulfosalicylic acid. After filtration, filtrate (2 mL) was treated with acid-ninhydrin reagent and boiled for 1 h. Chromophore was extracted using toluene and absorbance was read at 520 nm. Amount of proline was calculated using the following formula:

(2) Proline μmoles/g  tissue=μg  proline/ml∗ml  of  toluene∗5115.5∗g  of  sample.

#### 2.2.4. Estimation of Total Phenolics (TP)

For the quantification of total phenolics, chilli seed powder (300 mg) was 5 mL of 80% methanol. To 1 mL of extract, total phenolics were estimated by adding 0.5 mL Folin-Ciocalteu reagent, 7.5 mL ddH_2_O, followed by addition of 1.5 mL of 20% sodium carbonate. Absorbance was read at 755 nm with spectrophotometer. Estimation of total phenolics (mg/g) was measured as described by Tohma et al. [23].

#### 2.2.5. Extraction and Estimation of Hydrogen Peroxide (H_2_O_2_)

For the quantification of hydrogen peroxide, 100 mg of chilli seed powder was extracted with 0.1% trichloroacetic acid. After centrifugation at 10,000 ×g for 5 min, supernatant was used for estimation of H_2_O_2_ content (*μ*moles/g) as elucidated by Kaur et al. [24].

#### 2.2.6. Extraction and Estimation of Malondialdehyde (MDA)

To determine MDA content, chilli seed powder (100 mg) was extracted using 5 mL of 5% trichloroacetic acid. MDA was then measured using thiobarbituric acid reaction. Malondialdehyde (mmoles/g) was estimated as described by Rasool et al. [25].

### 2.3. Imposition of Drought Stress

Seeds of twenty genotypes were sown in black trays. After 30 days, seedlings were transplanted into pots. Moisture stress was imposed one week after transplantation at vegetative phase. A completely randomized block design (CRD) was performed with three replications, where seedlings of each variety were independently assigned to three water regimes: 100% Field Capacity (FC), control; 80% FC, moderate stress; and 60% FC, severe stress for one week using gravimetric method [26] at K L University fields, Vaddeswaram, Andhra Pradesh, India, in the years 2016-2017. This method involves quantification of water used by individual plant gravimetrically by weighing pots manually twice a day followed by replacing the water transpired to maintain the respective moisture stress conditions.

### 2.4. Drought Tolerance Indices

#### 2.4.1. Relative Water Content (RWC)

Relative water content of leaves was measured by using the method as described by Ali and Ashraf [27]. Fully expanded leaves were excised and fresh weight was recorded. Later leaves were soaked in distilled water at room temperature for 3 h and its turgid weight was recorded. Dry weight of leaves was documented after drying the leaves at 70°C for 48 h. RWC of leaves was then calculated using the following formula:

(3) $$\begin{array}{c} RWC\;\;\left(\;\%\;\right)=\frac{Fresh\;\;weight-DryWeight}{Turgid\;\;Weight-Dry\;\;Weight}*100. \end{array}$$

#### 2.4.2. Total Chlorophyll Content

Leaf discs were incubated in acetone and dimethyl sulphoxide (DMSO) in 1 : 1 proportion for 6–8 h. After incubation, optical density of the extract is measured at 645 nm and 663 nm using UV-visible spectrophotometer. Total chlorophyll content was derived according to method described by Mafakheri et al. [28].

#### 2.4.3. Assay for Superoxide Dismutase (SOD) Activity

Control and treated Leaf samples (0.5 g) were homogenized with 3.0 ml of potassium phosphate buffer and centrifuged at 2000 rpm for 10 min. To 0.2 ml of enzyme, 1.2 ml of sodium pyrophosphate buffer (0.0.25 M, pH-8.3), 0.1 ml of phenazine methosulfate (186 *μ*M), 0.3 ml of nitro blue tetrazolium (NBT) (300 *μ*M), and water in a volume of 2.8 ml were added. Reaction was initiated by adding 0.2 ml of NADH (780 *μ*M). The reaction mixture was incubated at 30°C for 90 s. Then, 1.0 ml of glacial acetic acid was added to stop the reaction. Followed by reaction culmination, the reaction mixture was then shaken with 4.0 ml of n-butanol and then allowed to stand for 10 min and centrifuged. The intensity of chromogen in the butanol layer was read at 560 nm in a spectrophotometer. One unit of SOD enzyme activity was defined as the amount of enzyme that gave 50% inhibition of reduction of NBT [29].

#### 2.4.4. Assay for Catalase (CAT) Activity

Leaf samples from control and treated plants (0.5 g each) were homogenized using 5 ml of 50 mM potassium phosphate buffer containing 0.1 mM EDTA. The solution was centrifuged at 12,000 rpm for 10 min at 4°C. In the experimental cuvette, H2O2-phosphate buffer (3.0 ml) was taken followed by addition of 40 *μ*l of enzyme extract and rigorous mixing was done. Catalase activity was quantified by decomposition of H_2_O_2_ which was measured according to the method described by Huseynova [30].

DPPH radical scavenging activity, reducing power, proline content, total phenolics, and H_2_O_2_ and MDA contents in seedlings were quantified using leaf samples (1 g) from control and treated seedlings according to methods cited above.

### 2.5. Statistical Analysis

All samples were collected in triplicate and data were analyzed with One-Way ANOVA. CD values were determined using OPSTAT-HAU, Hisar, India. Data matrix containing 20 rows (samples) and 16 columns (variables), six from seeds and ten from seedlings, was built. Principal component analysis (PCA) was pattern recognition technique used in this work. PCA allows exploring and analyzing the data structure, relationship between samples, variables, and correlations between variables [31]. PCA was done using XLSTAT.

## 3. Results

### 3.1. Seed Antioxidative Potential

#### 3.1.1. Total Phenolics, Proline, Hydrogen Peroxide, and Malondialdehyde Content

In the present study, average TP was found to be 0.33 mg/g (Table 2). Among the genotypes KCa-5 was highest (0.55 mg/g) followed by KCa-10 (0.41 mg/g), KCa-7 (0.40 mg/g), and KCa-17 (0.38 mg/g) TP while KCa-1 and KCa-3 were the lowest (0.28 mg/g). Proline content among the genotypes ranged from 0.13 *μ*moles/g to 1.69 *μ*moles/g (Table 2). KCa-5 and KCa-1 possessed high (1.69 *μ*moles/g) and low (0.13 *μ*moles/g) proline content. Genotypes KCa-10 (0.84 *μ*moles/g), KCa-7 (0.76 *μ*moles/g), KCa-17 (0.68 *μ*moles/g), and KCa-8 (0.68 *μ*moles/g) also possessed above average proline content.

Mean H_2_O_2_ content among the genotypes under study was 167.93 *μ*moles/g. Genotypes KCa-3 (227.17 *μ*moles/g) followed by KCa-11 (216.38 *μ*moles/g), KCa-18 (214.05 *μ*moles/g), and KCa-4 (199.08 *μ*moles/g) were observed to have maximum accumulation of hydrogen peroxide while KCa-5 genotype had lowest (117.38 *μ*moles/g) accumulation (Table 2). Mean MDA content among the hot pepper genotypes was observed to be 7.56 mmoles/g. Besides KCa-12 (14.52 mmoles/g), genotypes KCa-4 (13.42 mmoles/g) and KCa-8 (13.27 mmoles/g) showed high amounts of MDA while KCa-5 (0.83 mmoles/g) along with KCa-7 (1.52 mmoles/g) and KCa-10 (2.26 mmoles/g) showed lowest amounts of MDA (Table 2).

#### 3.1.2. DPPH Radical Scavenging Assay and Reducing Power Assay

DPPH radical scavenging activity ranged from 11.43% to 79.12% (Table 3). Among the genotypes, KCa-5 had highest while KCa-1 had least percentage of DPPH radical scavenging activity. Reducing power ability among twenty chilli genotypes was 49.53%. Meanwhile, KCa-10 exhibited highest (76.13%) reducing power, followed by KCa-5 (73.1%), and KCa-1 was observed to possess lowest (15.77%) percentage of reducing power (Table 3). In seeds, genotypes revealed significant correlation (0.88) between percentage of DPPH radical scavenging activity and total phenolics. Negative correlation was observed between total phenolics and H_2_O_2_ (−0.42) (Table 4).

### 3.2. Drought Tolerance Indices in Seedlings

#### 3.2.1. Total Phenolics, DPPPH Radical Scavenging Activity, and Reducing Power

**Figure 1:**
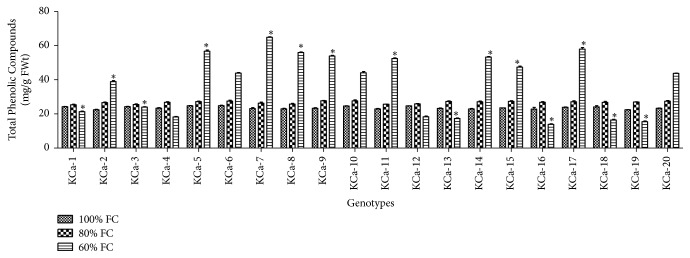
Total phenolics in seedlings of twenty hot pepper genotypes at control and stress conditions. Data mentioned is mean values of three replicates ± SD. *∗* indicates significant difference within 60% FC among genotypes at 0.01 level of probability.

TP at 100% FC ranged from 22.43 to 24.83 mg/g FWt (Figure 1). It has increased by 0.13-fold and 0.60-fold at moderate and severe water stress, respectively. At 60% FC, the increase in TP was trivial and ranged from 25.39 to 27.69 mg/g FWt (Figure 1). Average TP was 37.90 mg/g FWt at 60% FC. Genotype KCa-7 (64.91 mg/g FWt) was observed to have high TP, while KCa-16 (13.88 mg/g FWt) showed low TP (Figure 1).

**Figure 2:**
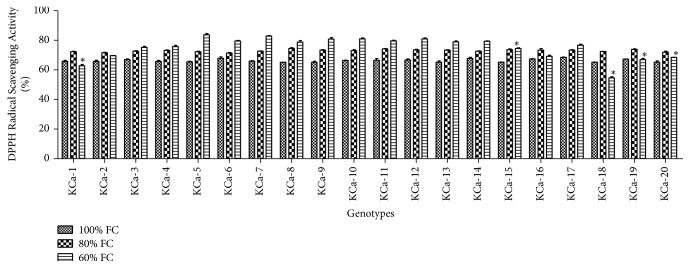
DPPH radical scavenging activity in seedlings of twenty hot pepper genotypes at control and stress conditions. Data mentioned is mean values of three replicates ± SD. *∗* represents significant difference within 60% FC among genotypes at 0.01 level of probability.

**Figure 3:**
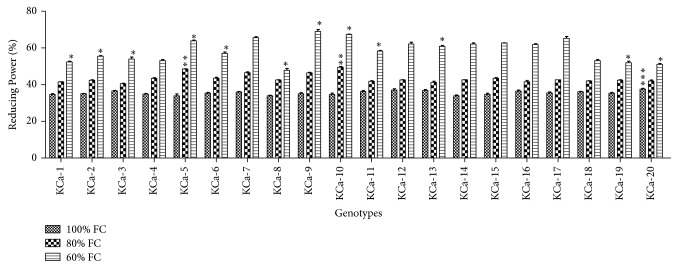
Reducing power in seedlings of twenty hot pepper genotypes at control and stress conditions. Data mentioned is mean values of three replicates ± SD. *∗∗∗* denotes significant difference within 100% FC, *∗∗* denotes significant difference within 80% FC, and *∗* denotes significant difference within 60% FC among genotypes at 0.01 level of probability.

Under control conditions, DPPH radical scavenging activity ranged from 65.13 to 68.47% (Figure 2). Scavenging activity was increased by 0.1-fold when plants were rendered to 80% FC. At severe stress, the scavenging activity was increased by 0.13-fold. Highest DPPH radical scavenging activity was possessed by KCa-5 (83.73%) followed by KCa-7 (82.85%) (Figure 2). Reducing power ranged between 33.89 and 37.54% when seedlings were grown at 100% FC (Figure 3). Reducing power was increased by 0.22-fold and 0.65-fold in seedlings grown at 80% and 60% FC, respectively (Figure 3).

#### 3.2.2. Proline, Hydrogen Peroxide, and Malondialdehyde Contents

**Figure 4:**
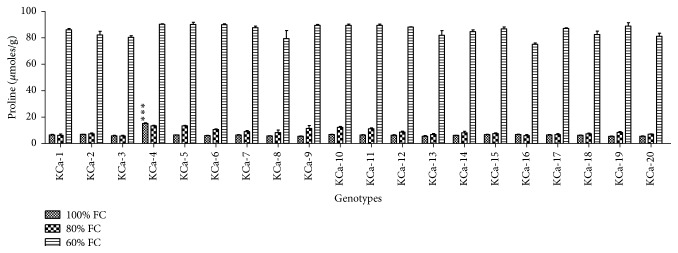
Total proline content in seedlings of twenty hot pepper genotypes at control and stress conditions. Data mentioned is mean values of three replicates ± SD. *∗∗∗* denotes significant difference within 100% FC.

At 100% FC, average proline content was observed to be 6.68 *μ*moles/g (Figure 4) and was increased to 32% in seedlings at 80% FC. At moderate stress, lowest proline content was observed in KCa-3 (5.70 *μ*moles/g) and highest proline content was found in KCa-4 (13.41 *μ*moles/g) followed by KCa-5 (1.38 *μ*moles/g) (Figure 4). There was 11.80-fold increase in proline accumulation in the seedlings when rendered to severe stress. Genotype KCa-16 was observed to have low proline accumulation (75.14 *μ*moles/g) whereas highest accumulation of proline was observed in KCa-4 (90.30 *μ*moles/g) (Figure 4).

**Figure 5:**
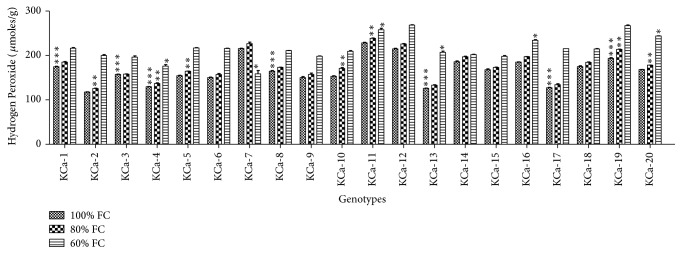
Hydrogen peroxide content in seedlings of twenty hot pepper genotypes at control and stress conditions. Data mentioned is mean values of three replicates ± SD. *∗∗∗* indicates significant difference within 100% FC, *∗∗* indicates significant difference within 80% FC, and *∗* indicates significant difference within 60% FC among genotypes at 0.01 level of probability.

**Figure 6:**
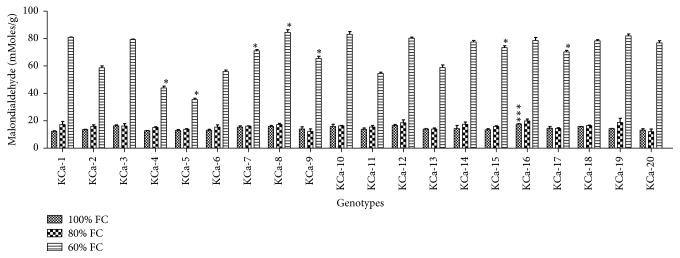
Malondialdehyde content in seedlings of twenty hot pepper genotypes at control and stress conditions. Data mentioned is mean values of three replicates ± SD. *∗∗∗* indicates significant difference within 100% FC and *∗* indicates significant difference within 60% FC among genotypes at 0.01 level of probability.

Average H_2_O_2_ content at 100% FC was 166.95 *μ*moles/g ranging from 117.63 to 228.38 *μ*moles/g (Figure 5). This has been increased by 5-fold and 29-fold when plants were subjected to 60% FC and 40% FC correspondingly. At 60% FC, high production of H_2_O_2_ was observed in KCA-11 (238.73 *μ*moles/g) and less content was observed in KCa-2 (125.72 *μ*moles/g). Under severe stress conditions, genotype KCa-12 was observed to have higher accumulation of H_2_O_2_ (168.36 *μ*moles/g) while KCa-7 showed lower accumulation of H_2_O_2_ (158.6 *μ*moles/g) (Figure 5). Average Malondialdehyde content in hot pepper genotypes was 14.15 mmoles/g, 15.93 mmoles/g, and 69.46 mmoles/g when seedlings were subjected to 80% and 60% FC, respectively (Figure 6). The increase in the production of MDA was 0.10-fold.

#### 3.2.3. Relative Water Content and Chlorophyll Content

**Figure 7:**
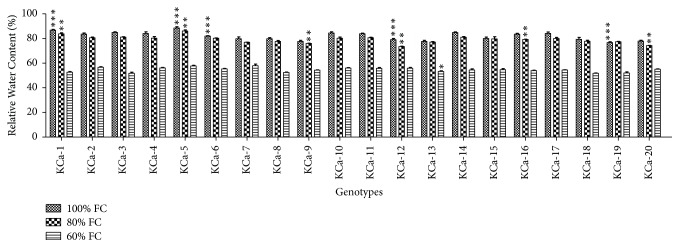
Relative water content in seedlings of twenty hot pepper genotypes at control and stress conditions. Data mentioned is mean values of three replicates ± SD. *∗∗∗* indicates significant difference within 100% FC, *∗∗* indicates significant difference within 80% FC, and *∗* indicates significant difference within 60% FC among genotypes at 0.01 level of probability.

RWC across the genotypes under normal conditions ranged from 77 to 87% (Figure 7). No significant decrease was observed in RWC when plants were subjected to moderate stress. Under severe water stress conditions, a decrease of 1.49-fold was observed in RWC. KCa-7 (57.92%) processed high RWC followed by KCa-5 (57.71%) and KCa-2 (56.64%). Among twenty hot pepper genotypes, KCa-3 (51.80%) retained lowest RWC (Figure 7).

**Figure 8:**
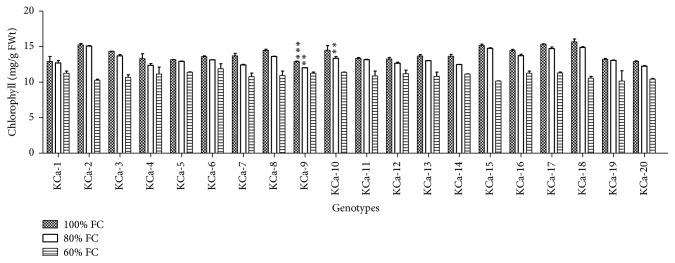
Total chlorophyll content in seedlings of twenty hot pepper genotypes at control and stress conditions. Data mentioned is mean values of three replicates ± SD. *∗∗∗* indicates significant difference within 100% FC and *∗∗* indicates significant difference within 80% FC.

At 100% FC total chlorophyll content across the genotypes was of 13.93 mg/g FW ranging from 13 to 16 mg/g FW (Figure 8). A nonsignificant decrease of 1.04-fold was observed at moderate stress while significant decrease of 1.27-fold was observed at severe moisture stress conditions. Among the genotypes subjected to severe stress, genotype KCa-6 (11.87 mg/g FW) retained high chlorophyll content while KCa-15 (10.16 mg/g FW) showed less chlorophyll content under moisture stress (Figure 8).

#### 3.2.4. Superoxide Dismutase and Catalase Activities

**Figure 9:**
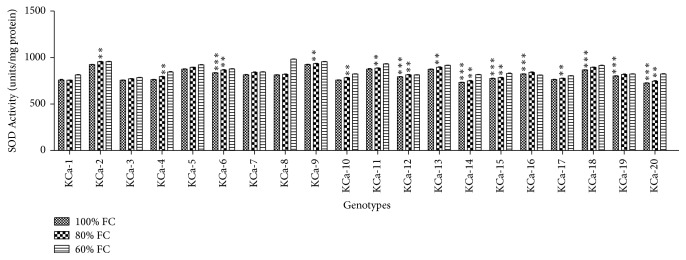
Activity of superoxide dismutase enzyme in seedlings of twenty hot pepper genotypes at control and stress conditions. Data mentioned is mean values of three replicates ± SD. *∗∗∗* indicates significant difference within 100% FC and *∗∗* indicates significant difference within 80% FC.

**Figure 10:**
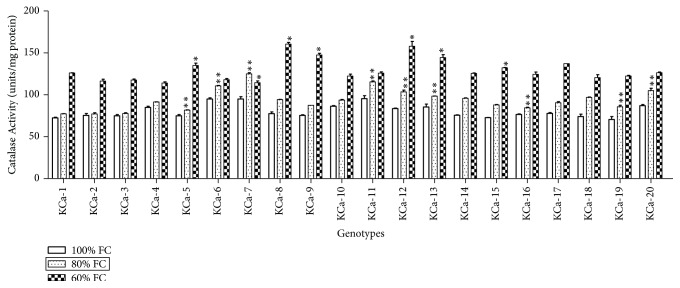
Activity of catalase enzyme in seedlings of twenty hot pepper genotypes at control and stress conditions. Data mentioned is mean values of three replicates ± SD. *∗∗* indicates significant difference within 80% FC and *∗* indicates significant difference within 60% FC among genotypes at 0.01 level of probability.

SOD activity of twenty hot pepper genotypes ranged from 725 to 924.5 units/mg protein when seedlings were grown under control conditions (Figure 9). At moderate stress, the average SOD activity among genotypes was increased by 2.24% and highest SOD activity was observed in KCa-2 (955 units/mg protein), followed by KCa-9 (934 units/mg protein). When seedlings were subjected to 60% FC, highest SOD activity was observed in KCa-8 (983.5 units/mg protein) and KCa-2 (956.5 units/mg protein) and low SOD activity was seen in KCa-3 (785.5 units/mg protein) (Figure 9). Catalase activity ranged between 70.5 and 95.5 units/mg protein (Figure 10). Catalase activity was increased by 16.73% and 60.72% when seedlings were rendered to 80 and 60% FC, respectively (Figure 10).

### 3.3. Principal Component Analysis

**Figure 11:**
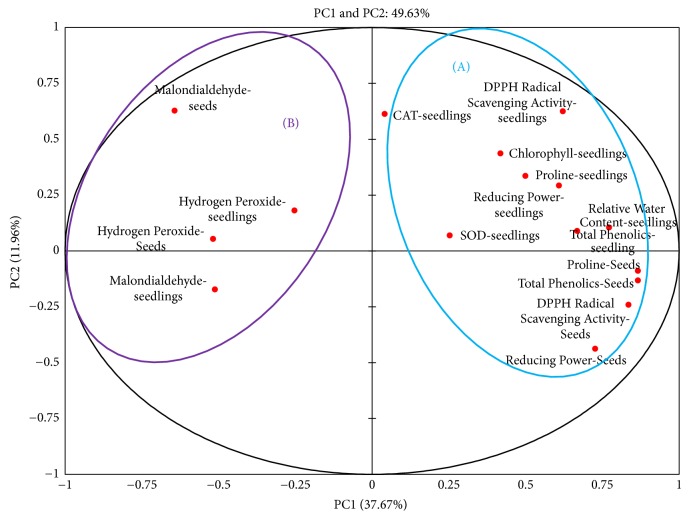
Principal component analysis of seed antioxidants and antioxidants, physiological, and biochemical parameters in seedlings among twenty hot pepper genotypes.

Correlation between seed antioxidants in seeds and drought tolerance traits were studied using principal component analysis (PCA). Among the sixteen principal components (PC1–PC16) obtained, PC1 and PC2 were used for analysis. PCA plot obtained for seed antioxidants and physiological and biochemical parameters for twenty genotypes was depicted (Figure 11). The measured variables Principal Component 1 (PC1) showed 11.96% and Principal Component 2 (PC 2) 37.67% and the total variance with a cumulative eigenvalue of 49.63%. It was evident that MDA and H_2_O_2_ were clustered together into group B on the left side of the plot and seed DPPH, total phenolics, reducing power, and proline and the drought tolerance indices of seedlings (DPPH, TP, proline, RWC, chlorophyll contents, SOD, and catalase activities) were clustered into group A towards the right side of plot (Figure 11). The traits clustered together are strongly correlated to each other.

**Figure 12:**
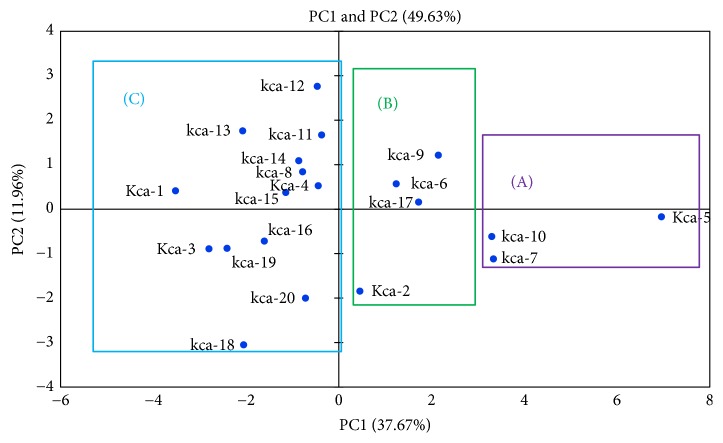
Principal component analysis of chilli genotypes.

PCA plot of the twenty genotypes was illustrated (Figure 12). PC1 and PC2 showed 37.67% and 11.96%, respectively, with a total variance with a cumulative eigenvalue of 49.63%. In PCA genotypes KCa-5, KCa-7, and KCa-10 which are observed to be tolerant were clustered together into group A (Figure 12). Genotypes KCa-2, KCa-6, KCa-9, and KCa-17 which were recognized to be moderately tolerant were clustered into group B. The rest of the genotypes were grouped into group C (Figure 12) and were observed to be susceptible.

## 4. Discussion

Like animals, plants are obligate aerobic organisms. As oxygen concentration is much higher in plants than in animals, plant tissues experience wide oxygen fluctuations when exposed to abiotic stress [32]. Also, due to natural oxygen metabolism in plants, they are likely to produce huge ROS continuously [33]. Repeated production of ROS creates an oxidative environment which negatively affects the redox balance in the cell. Altered redox state changes cellular signaling pathways and other metabolic processes. Plants possess an integrated system of enzymatic and nonenzymatic antioxidant defense system that controls ROS production by scavenging and thereby protecting the plant cells from oxidative damage [34].

Antioxidant potential of seeds is vital in determining the inherent stress tolerance in plants. As seed traits determine growth at vegetation period, seeds which show higher antioxidant properties would possess certain biochemical traits which would be utilized by seedlings to establish and survive under unfavorable conditions. Hence the present study focused on evaluating the impact of seed antioxidant properties on drought tolerance during vegetative phase in chilli. Plant phenolic compounds are potent antioxidants, which are capable of scavenging reactive oxygen species, thereby reducing the risk of oxidative damage. The antioxidant activity of the plant is mainly ascribed to the occurrence of phenolic compounds [35]. Genotypes KCa-5, KCa-10, and KCa-7 exhibited high total phenolics and also high DPPH scavenging activity. Genotypes KCa-1 and KCa-15 which have low phenolics exhibited the highest low DPPH scavenging activity. Wangcharoen and Morasuk [36] also reported significant correlation between DPPH radical scavenging assay and total phenolics. Also, antioxidant ability of seeds is positively correlated with phenolic compounds [37, 38].

Among the genotypes there was no significant variation in total phenolics and DPPH scavenging activity when seedlings were grown at 100% FC. When seedlings were subjected to 80% FC, there was trivial increase in TP and DPPH scavenging activity indicating a nonsignificant effect of moderate stress of hot pepper genotypes. Boutraa et al. [39] worked on drought tolerance in wheat cultivars using 80% FC as control. Genotypes demonstrating high DPPH scavenging activity as a result of high TP at seed stage continued to establish high TP resulting in high DPPH scavenging activity. Yet, KCa-5, which exhibited high total phenolics at 60% FC, showed less total phenolics at 80% FC. Also, DPPH scavenging activity was less in KCa-15 at seed level while it has been drastically increased at seeding stage when subjected to 80% FC. These differences indicate differential interaction of genotypes with different intensities of drought stress. According to Obidiegwu et al. [40], phenotypic responses of plants are often complex and are seasoned by interactive effects of plant genotype, duration of stress, and intensity of stress and on developmental stage at which stress has occurred. At moderate stress, genotypes KCa-6 and KCa-20 showed high TP but exhibited less DPPH scavenging activity. This is due to the fact that total antioxidant capacity did not depend on total phenolics and other attributed cellular components. According to Krishnan et al. [41] apart from phenolic compounds, flavanoid compounds and ascorbic acid content contribute to the total antioxidant activity. Also, in conjunction with nonenzymatic components, enzymatic component also comes into play to scavenge ROS at seedling or plant level [42].

Under stress conditions an excessive production of ROS causes oxidative damage which eventually leads to the plant death. A common effect of oxidative stress in biological membrane of plants was lipid peroxidation with MDA as one of its end products. Møller et al. [43] described that a valuable tool for measuring of oxidative lipid injury was estimation of MDA. From present investigation genotypes KCa-4, KCa-8, KCa-11, and KCa-12 were observed to possess accumulated levels of hydrogen peroxide and MDA which are subsequently attributed to elevated lipid peroxidation. When hot pepper seedlings were subjected to drought stress, there was significant increase in hydrogen peroxide and MDA. Intensity of hydrogen peroxide, consequently MDA, increased with the intensity of stress. Genotypes KCa-11, KCa-12, and KCa-16 had higher accumulation of H_2_O_2_ at both seed and seedling stage (80% FC and 60% FC). At moderate and severe stress, KCa-12 and KCa-16 exhibited higher lipid peroxidation which was evident from increased levels of MDA. But genotype KCa-11, at 60% FC, which has high amounts of H_2_O_2_ has lower lipid peroxidation. This can be attributed to high total phenolics in that genotype. Present investigation showed positive correlation between MDA and H_2_O_2_. Yang and Miao [44] reported parallel increase of MDA with increase in hydrogen peroxide when poplar species were submitted to progressive drought treatments. While genotypes KCa-5 and KCa-10 showing highest DPPH radical scavenging activity showed less hydrogen peroxide ensuring lowered lipid peroxidation. Ren et al. [45] elucidated lower membrane injury due to less accumulation of ROS in* Cerasus humilis*.

Accumulation of proline was considered to be a significant strategy employed by plants to achieve tolerance towards drought. Proline being an osmolyte helps the plant to maintain turgor pressure in the cell and allows the plant to survive under drought conditions by drawing extracellular water. According to Farooq et al. [46] cytosolic concentration of osmolytes is often increased under drought to maintain osmoregulation. It alleviates cytoplasmic acidosis. It also maintains appropriate NADP+/NADPH ratios compatible with metabolism [47]. Also, proline functions as molecular chaperons thereby stabilizing the structure of proteins. Proline accumulation buffers cytosolic pH and thus maintains redox status in cell. But not always the correlation between correlation between proline accumulation and abiotic stress tolerance is apparent. Proline is seen to accumulate both in sensitive plants and in tolerant plants [48]. Nonetheless several studies revealed that proline accumulation plays a significant role in conferring tolerance adverse environmental conditions [49, 50]. Under moisture stress, proline and RWC were considered to be important drought tolerance traits. Genotypes KCa-5, KCa-7, and KCa-10 containing high proline content in seeds gave better tolerance towards drought stress at seedling stage by retaining higher relative water contents thereby tolerating drought stress. According to Raymond and Smirnoff [51], the correlation between seed proline and stress tolerance would be due to the fact that proline accumulation takes place in roots due to its translocation from endosperm in the course of germination. Increased accumulation of proline in drought tolerant maize cultivars under progressive drought stress was reported by Anjum et al. [11]. Present results reveal that there exists correlation between proline and RWC among the genotypes and results were in accordance with that of Kaur et al. [24].

Chlorophyll content is an important drought tolerance trait which specifies photosynthetic efficiency under drought stress. Guo et al. [52] attributed decrease in chlorophyll content to decreased photosynthetic activity. Present study also revealed a negative correlation between drought intensity and chlorophyll content, and also Pirzad et al. [53] reported in seedlings of* Matricaria chamomilla* that chlorophyll concentration got reduced with increase in drought stress. Furthermore, genotypes KCa-3, KCa-11, and KCa-15 with higher hydrogen peroxide content possessed lowered levels of chlorophyll indicating the effect of ROS on membrane integrity. Zhao et al. [54] studied temperature induced changes in lipid peroxidation and chlorophyll in cucumber leaves.

Water stress inevitably coupled to oxidative stress resulting from enhanced accumulation of ROS. Activation and induction of antioxidant enzyme activities are general strategy adopted by the plant to combat oxidative stress. SOD is one of the key enzymes produced against oxidative stress for cellular production. According to Alscher et al. [55], ability of plants to overcome oxidative stress relies on activation of SOD activity followed by subsequent upregulation of other antioxidant enzymes. At 80% FC, SOD activity has been increased in all the genotypes but the increase is trivial. When the plants were subjected to mild and/or moderate drought stress, they exhibited increasing activities of antioxidant enzymes [56]. The activity of SOD enzyme further increased in all genotypes at 40% FC except in KCa-12 and KCa-16. Increase in SOD activity can be associated with increased production of active oxygen species. Increased SOD activity in response to drought stress has been reported in tomato [57], sunflower [58], poplar [59], cowpea [60], and liquorice [61]. The decreased activity of SOD in KCa-12 and KCa-16 can be attributed to the inactivation of enzyme under prolonged and increased drought stress. Abedi and Pakniyat [29] reported decreased SOD activity in oil seed rape under drought stress.

SOD activity results in the formation of H_2_O_2_ which is to be scavenged rapidly by antioxidant system [62]. Hence, often enhanced action of SOD is accompanied by enhanced H_2_O_2_ scavenging mechanisms like CAT. It is considered one of the important antidrought strategies to cope with oxidative stress. In the present investigation CAT activity is increased with increase in intensity of drought stress. Our results are in accordance with Bian and Jiang [63] who showed increase in CAT activity with stress. Also, the results are not in correlation with Abedi and Pakniyat [29] as they reported decreased CAT activity in increased stress. Declined CAT activity is considered as general response to different abiotic stresses [64]. According to Chaparzadeh et al. [65], CAT activity changes with stress duration, intensity of stress, and plant developmental stage.

In the present work PCA was used to describe the correlation between seed antioxidant properties and drought tolerance traits. According to Kim et al. [66], among multivariate methods, PCA is a frequently used method to classify samples. Analysis revealed strong correlation between different antioxidant properties of seeds and drought tolerance traits at vegetative phase using PC1 and PC2. Chunthaburee et al. [67] also reported correlation explained by first PC, as other PCs cover only little information of data sets. PCA clearly separated drought susceptible genotypes from drought tolerant genotypes. Mazid et al. [68] also clustered 41 different rice genotypes using PCA.

In a brief conclusion our study identified that genotypes Kca-5, Kca-7, and KCa-10 as drought tolerant while it identified KCa-3, Kca-8 KCa-11, Kca-13, and KCa-15 as susceptible. Identified genotypes can be used as parents in chilli hybrid breeding program. This approach will pave a way to screen the chilli genotypes against water stress tolerance for future breeding programme.

## Figures and Tables

**Table 1:** List of chilli genotypes used in the present work.

S. number	Genotypes (code)	Name of genotypes	Region cultivated
1	KCa-1	AC3-1-1-1	High altitude and tribal areas
2	KCa-2	LCA334-1-1-1-1	Krishna Godavari Zone
3	KCa-3	VM1-1-1-1	High altitude and tribal areas
4	KCa-4	LCA353-1-1	Krishna Godavari Zone
5	KCa-5	SHP4884-1-1	Northern Telangana Zone
6	KCa-6	BSS355-1-1-1-1	Krishna Godavari Zone
7	KCa-7	Devanur Deluxe-1-1-1	Krishna Godavari Zone
8	KCa-8	G4-1-1-1-1	Krishna Godavari Zone
9	KCa-9	CA960-1-1-1-1	Northern Telangana Zone
10	KCa-10	Indam05-1-1	Krishna Godavari Zone
11	KCa-11	Pusa Jwala-1-1-1	Krishna Godavari Zone
12	KCa-12	US341-1-1-1-1	Krishna Godavari Zone
13	KCa-13	Vishnu-1-1-1-1	Krishna Godavari Zone
14	KCa-14	AC1-1-1-1	High altitude and tribal area
15	KCa-15	VM2-1-1-1-1	High altitude and tribal area
16	KCa-16	HPH1048-1-1-1-1	Northern Telangana Zone
17	KCa-17	AC2-1-1-1-1	High altitude and tribal areas
18	KCa-18	Teja-1-1-1-1	Krishna Godavari Zone
19	KCa-19	Super10-1-1-1-1	Krishna Godavari Zone
20	KCa-20	Rabi222-1-1-1-1	Krishna Godavari Zone

**Table 2:** Total phenolics, proline, hydrogen peroxide (H_2_O_2_), and Malondialdehyde (MDA) in seeds of hot pepper.

Genotypes	Total phenolics (mg/g)	Proline (*µ*moles/g)	H_2_O_2_ (*µ*moles/g)	MDA (mMoles/g)
KCa-1	0.28^a^ ± 0.10	0.13^a^ ± 0.01	153.39 ± 0.71	11.96^q^ ± 0.56
KCa-2	0.31 ± 0.14	0.39 ± 0.01	129.23 ± 0.48	4^f^ ± 0.50
KCa-3	0.28 ± 0.03	0.27 ± 0.02	227.17^t^ ± 0.18	7.53^j^ ± 1.08
KCa-4	0.29 ± 0.10	0.40 ± 0.01	199.08 ± 0.39	13.42 ± 0.82
KCa-5	0.55^t^ ± 0.03	1.69^t^ ± 0.10	117.38^a^ ± 0.60	0.83^a^ ± 0.07
KCa-6	0.29 ± 0.17	0.37 ± 0.05	125.33^b^ ± 0.58	4.16 ± 0.45
KCa-7	0.4 ± 0.10	0.76 ± 0.03	174.53 ± 0.34	1.52 ± 0.54
KCa-8	0.33 ± 0.08	0.68 ± 0.01	181.21^n^ ± 0.85	13.27^r^ ± 0.90
KCa-9	0.35 ± 0.05	0.38 ± 0.05	148.11^f^ ± 0.72	5.81^i^ ± 0.41
KCa-10	0.41 ± 0.03	0.85 ± 0.01	126.30 ± 1.28	2.26^c^ ± 0.30
KCa-11	0.28 ± 0.01	0.38 ± 0.02	216.38 ± 0.47	11 ± 1.00
KCa-12	0.36 ± 0.03	0.4 ± 0.01	187.69 ± 0.96	14.52 ± 0.49
KCa-13	0.29 ± 0.05	0.16 ± 0.01	168.87 ± 1.67	10.57 ± 0.82
KCa-14	0.29 ± 0.04	0.2 ± 0.02	173.73 ± 0.23	10.13 ± 0.59
KCa-15	0.28 ± 0.05	0.34 ± 0.01	187.37 ± 0.36	9.64^k^ ± 0.57
KCa-16	0.31 ± 0.05	0.37 ± 0.01	168.59^i^ ± 0.57	9.85 ± 0.88
KCa-17	0.38 ± 0.03	0.69^p^ ± 0.01	156.50 ± 0.62	4.13 ± 0.02
KCa-18	0.34 ± 0.03	0.24 ± 0.015	214.05^r^ ± 0.86	3.24^e^ ± 0.86
KCa-19	0.30 ± 0.02	0.39 ± 0.01	128.77 ± 0.83	11 ± 1.31
KCa-20	0.33 ± 0.01	0.4 ± 0.01	174.94 ± 1.85	2.42 ± 1.05
CD	0.06	0.26	6.18	0.72

The data shown are mean of three replicates ± standard deviation. Within the column means followed by different letters are significantly different with other genotypes at 0.05 level of probability.

**Table 3:** DPPH scavenging activity and reducing power in seeds of twenty chilli genotypes.

Genotypes	DPPH radical scavenging activity (%)	Reducing power (%)
KCa-1	11.43^a^ ± 0.09	15.77^a^ ± 0.44
KCa-2	44.2^n^ ± 0.48	53.7^j^ ± 0.49
KCa-3	33.12 ± 1.01	32.82 ± 1.23
KCa-4	34.47 ± 0.46	51.73^i^ ± 0.65
KCa-5	79.12^t^ ± 0.49	73.1^s^ ± 0.59
KCa-6	35.77^i^ ± 1.06	55.55^l^ ± 0.62
KCa-7	51.28^r^ ± 0.41	70.05^r^ ± 1.54
KCa-8	37.81 ± 0.86	61.64 ± 0.47
KCa-9	46.56 ± 0.70	68.71 ± 0.26
KCa-10	59.13^s^ ± 0.09	76.13^t^ ± 0.94
KCa-11	34.39 ± 0.22	34.81 ± 0.20
KCa-12	49.36^q^ ± 0.46	32.33^e^ ± 0.37
KCa-13	18.84^b^ ± 0.56	17.03^b^ ± 0.53
KCa-14	32.51^c^ ± 0.46	27.98^c^ ± 0.02
KCa-15	32.6 ± 0.47	31.28^d^ ± 0.89
KCa-16	38.75^l^ ± 0.83	54.82 ± 0.17
KCa-17	37.76 ± 0.36	68.49^p^ ± 0.54
KCa-18	45.95^o^ ± 0.03	60.49^n^ ± 1.19
KCa-19	34.31 ± 1.17	45.12^h^ ± 0.86
KCa-20	41.79^m^ ± 0.86	59.13^m^ ± 0.06
CD	0.62	0.70

The data shown are mean of three replicates ± standard deviation. Within the column means followed by different letters are significantly different with other genotypes at 0.05 level of probability.

**Table 4:** Correlations between antioxidant capacities and total phenolics, H_2_O_2_, and MDA in hot pepper seed extracts.

S. number	Correlation	*R* value	*R* ^2^ value
1	DPPH versus total phenolics	0.884338	0.782054
2	Reducing power versus total phenolics	0.661986	0.438226
3	Total phenolics versus MDA	−0.58839	0.346208
4	Total phenolics versus H_2_O_2_	−0.42375	0.179568

*R*—correlation coefficient; *R* ^2^—coefficient of determination.
